# Anti-PD-1 mAb pre-radiotherapy (RT) loading dose and fractionated RT induce better tumor-specific immunity and tumor shrinkage than sequential administration in an HPV+ head and neck cancer model

**DOI:** 10.1186/2051-1426-3-S2-P314

**Published:** 2015-11-04

**Authors:** Raghvendra M Srivastava, David A Clump, Robert L Ferris

**Affiliations:** 1 University of Pittsburgh Cancer Institute, Pittsburgh, PA, USA; 2 UPMC Department of Radiation Oncology, Pittsburgh, PA, USA; 3 Department of Otolaryngology and Cancer Immunology Program, University of Pittsburgh Cancer Institute, Pittsburg, PA, USA

## 

Radiotherapy (RT) is a standard therapeutic strategy in the treatment in head and neck cancer (HNC), but many patients still experience recurrence and metastasis. Interestingly, radiotherapy (RT) may also induce immunomodulatory effects. Given the recent, exciting responses seen using anti-PD-1 (programmed death-1) checkpoint blockade immunotherapy in recurrent/metastatic disease, including HNC, we evaluated the combination of RT with anti-PD-1 therapy in a pre-clinical mouse model of locally advanced, untreated HPV-positive HNC. We compared utilizing PD-1 blockade before, during or after RT, as well as whether a single large faction (12Gy) or multiple smaller RT doses (2 Gy X 10 fractions) confers optimal antitumor immune responses and tumor shrinkage. We observed that fractionated doses of RT induced the highest PD-L1 (programmed death-ligand 1) expression on HNC cells in vitro and in treated mice. A loading dose of anti-PD-1 mAb therapy prior to RT appeared to be important for best therapeutic outcome, with greatest tumor response and tumor-specific immunity using PD-1 Ab loading dose than sequential administration of anti-PD-1 mAb after fractionated RT (p < 0.0001). Expression and intensity of PD-1 receptor expression on circulating T cells differentially impacted the T cell phenotype and anti-tumor outcome, with loss of PD-1(high) exhausted T cells during the best tumor response (p < 0.05). The combination of fractionated RT + anti-PD-1 Ab optimally upregulated the frequency of HPV E7 tumor antigen-specific T cells (p < 0.05). This study may facilitate strategies required for the combination of RT and immune checkpoint inhibitor in clinical trials, enabling more effective clinical activity and biomarker evaluation.

